# A Hierarchical Sensor Data Fusion and Roving Sensor Network Framework for Structural Health Monitoring: Application to Bridge Retrofitting

**DOI:** 10.3390/s26113597

**Published:** 2026-06-05

**Authors:** Emrullah Dar, Tarık Tufan, Selahattin Akalp, Ferit Yardımcı

**Affiliations:** 1 Yapi Checkup Software Co., Ltd., Yenişehir, 33110 Mersin, Türkiye; 2 Department of Civil Engineering, Istanbul Medeniyet University, 34700 Istanbul, Türkiye; tarik.tufan@medeniyet.edu.tr; 3 Department of Civil Engineering, Batman University, 72100 Batman, Türkiye; selahattin.akalp@batman.edu.tr; 4 Department of Civil Engineering, Turkish-German University, 34820 Istanbul, Türkiye; yardimci@tau.edu.tr

**Keywords:** structural health monitoring, roving sensors, block-wise PCA, sensor fusion, vibration sensing, signal processing, environmental and operational variations (EOV), ARX model, wavelet packet decomposition, damage detection

## Abstract

Extracting reliable damage-sensitive features from sparse sensor networks under Environmental and Operational Variations (EOV) remains a critical challenge in Structural Health Monitoring (SHM). The purpose of this study is to overcome this limitation by proposing a novel, data-driven framework utilizing a cost-effective network of high-sensitivity triaxial roving accelerometers. The methodology integrates an AutoRegressive with eXogenous inputs (ARX) model and Wavelet Packet Decomposition (WPD) to extract robust, damage-sensitive features from complex vibration data. To handle the high-dimensionality of the extracted signals and achieve optimal multi-sensor data fusion, Block-wise Principal Component Analysis (PCA) is employed as a signal sanitation and feature reduction tool. This algorithmic pipeline is applied to a full-scale bridge pier subjected to RC jacketing. The structural enhancements and dynamic behavior shifts post-retrofitting were statistically quantified using the Mahala Nobis distance. The analysis revealed a 41.2% attenuation in median vibration intensity and successfully verified the structural improvements at a 99% confidence interval, clearly distinguishing the retrofitting effects from ambient noise. The proposed framework successfully isolates true structural changes from EOV, providing a reliable non-destructive evaluation tool for continuous monitoring in practical civil engineering applications.

## 1. Introduction

The continuous aging of civil infrastructure necessitates robust Structural Health Monitoring (SHM) systems [1,2]. Recent advancements in SHM have facilitated a transition from periodic manual inspections to continuous, data-driven diagnostic systems, enabling real-time condition assessment of critical transport infrastructures [3]. The primary technical challenge lies in developing cost-effective and scalable sensing strategies that can deliver high-fidelity diagnostic data under complex operational conditions. To restore structural integrity, localized retrofitting interventions, such as the RC jacketing of specific bridge piers, are widely implemented [4]. However, quantifying these structural shifts using a sparse sensor network remains a significant sensing and signal processing challenge, as localized stiffening alters the global dynamical topology in ways that are difficult to capture with limited measurement points [5].

Traditionally, Operational Modal Analysis (OMA) has served as the primary method for vibration-based SHM, relying heavily on tracking shifts in global modal parameters, primarily natural frequencies [6,7]. Nevertheless, there is a diverging consensus regarding the reliability of frequency-shift methods [8]. Global natural frequencies are generally known to be insensitive to localized stiffness variations. Furthermore, minor frequency-domain features are often buried within signal noise induced by Environmental and Operational Variations (EOVs), such as daily temperature gradients. Consequently, advanced denoising and autonomous feature extraction techniques are required to isolate true structural changes [9,10]. Therefore, observing a slight frequency increase post-retrofit is insufficient to comprehensively verify global structural stabilization. To address the limitations of frequency-only monitoring, the focus must shift towards spatial dynamic interactions, such as cross-span signal coherence and spatial decoupling [11]. Proving both direct decoupling at the intervened joint and indirect decoupling—the cessation of dynamic interaction between adjacent unretrofitted spans—remains a complex task that conventional linear modal analysis struggles to reliably quantify [12].

Traditional SHM sensing architectures have historically relied on dense, permanently installed sensor arrays to achieve adequate spatial resolution. However, scaling these dense networks for large continuous civil infrastructures introduces prohibitive hardware costs, extensive cabling or wireless bandwidth requirements, and long-term maintenance challenges [13]. In contrast, roving sensor technology—where a limited number of high-fidelity sensors are sequentially deployed across multiple measurement setups—presents a highly cost-effective and scalable alternative. This strategy offers high-resolution spatial mapping of structural dynamics with minimal hardware investments [14]. Recent studies have further demonstrated the viability of output-only system identification techniques utilizing limited roving sensors to monitor full-scale bridges under random traffic loads [15]. Concurrently, advancements in multi-sensor data fusion have significantly enhanced the reliability of SHM frameworks by integrating diverse structural responses into cohesive damage indices [16]. Nevertheless, the primary bottleneck of sparse and roving sensing lies in the signal processing domain; the sequential nature of data acquisition inherently produces fragmented, non-synchronous datasets. To extract meaningful global condition indicators from such setups without relying on a fixed reference sensor, the challenges of non-synchronous data fusion must be explicitly addressed through robust, autonomous algorithmic pipelines [17].

Due to the limitations of traditional modal parameters, the SHM community has increasingly adopted data-driven Machine Learning (ML) techniques [18,19]. Time-series modeling, such as Autoregressive with eXogenous inputs (ARX) models, has proven highly effective in extracting latent interaction dynamics directly from raw ambient data [20]. Concurrently, advanced signal processing tools like Wavelet Packet Decomposition (WPD) capture the spectral energy topology of non-stationary structural responses [21]. Despite these advancements, most existing data-driven SHM frameworks rely on a single feature domain, which limits the capacity of algorithms to fully capture complex structural regime shifts. Therefore, there is a critical gap in the literature regarding the implementation of hierarchical multi-view feature fusion to evaluate structural retrofitting.

To address this limitation, the primary aim of this study is to propose a novel, data-driven framework. The primary contribution of this study is the architectural fusion of multi-view feature domains, rather than the introduction of a new mathematical operator. This framework addresses a persistent civil engineering challenge: quantifying localized structural changes using sparse, non-synchronous roving sensors without requiring global time synchronization or mode shape reconstruction. The proposed methodology integrates Interaction Dynamics, Spectral Topology, and Statistical Complexity into a unified latent space using Block-wise Principal Component Analysis (PCA) [22,23,24]. Furthermore, the Mahalanobis distance is employed to statistically quantify spatial decoupling, transforming the latent shift into a robust condition index [2]. The principal conclusions of this study demonstrate that localized RC jacketing leads to a significant global structural regime shift. The proposed multi-view fusion explicitly visualizes a zero-overlap bifurcation between the pre- and post-retrofit states. This demonstrates that localized stiffening induces both direct and indirect spatial decoupling across the bridge structure, supported by a 99% statistical confidence level.

## 2. Materials and Methods

This section describes the deployment of a sparse sensor network on a full-scale bridge testbed and the subsequent mathematical formulation of the proposed hierarchical multi-sensor data fusion framework. To ensure reproducibility, all hardware constraints, data acquisition protocols, and signal processing parameters are explicitly defined. The ambient vibration data were collected using EQMet TSA-SMA accelerometers (EQMet, Pasadena, CA, USA), and all signal processing and algorithmic computations were performed using MATLAB 2026a (MathWorks, Natick, MA, USA). The raw vibration datasets generated during the ambient vibration tests are available from the corresponding author upon reasonable request. No generative artificial intelligence (GenAI) technologies were utilized in the study design, data collection, or algorithmic computations presented herein.

### 2.1. Description of the Bridge Structure and Retrofit Strategy

**Figure 1:**
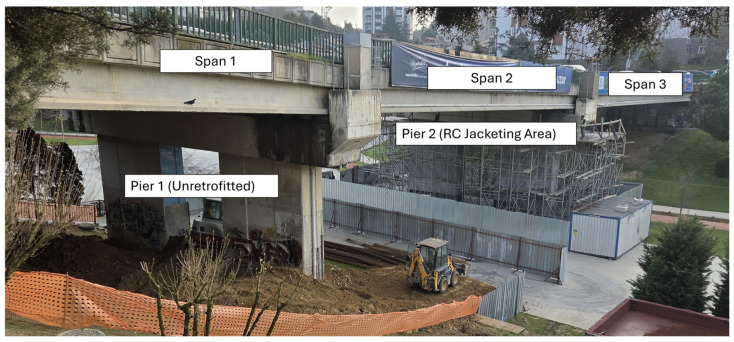
General overview of the multi-span bridge during the retrofitting phase. The photograph highlights the unretrofitted reference pier (Pier 1) alongside the localized structural intervention zone, where reinforced concrete (RC) jacketing was exclusively applied to Pier 2.

The physical asset investigated in this study is a continuous multi-span reinforced concrete (RC) highway bridge, as depicted in Figure 1. Preliminary structural assessments revealed localized degradation and dynamic looseness at the interface of Pier 2 (located between Span 2 and Span 3). To restore structural integrity, a localized retrofitting strategy was implemented via RC jacketing strictly around Pier 2. This strengthening technique significantly increases the axial and shear capacities, as well as the flexural stiffness of the treated member [25]. No structural modifications were made to the adjacent Pier 1 or the bridge deck.

The geometric and material properties of the structure, alongside the exact nature of the retrofitting intervention, have been detailed based on the official structural analysis and as-built project documentation. These engineering parameters are explicitly summarized in Table 1.

### 2.2. Sensor Deployment and Data Acquisition Campaigns

**Figure 2:**
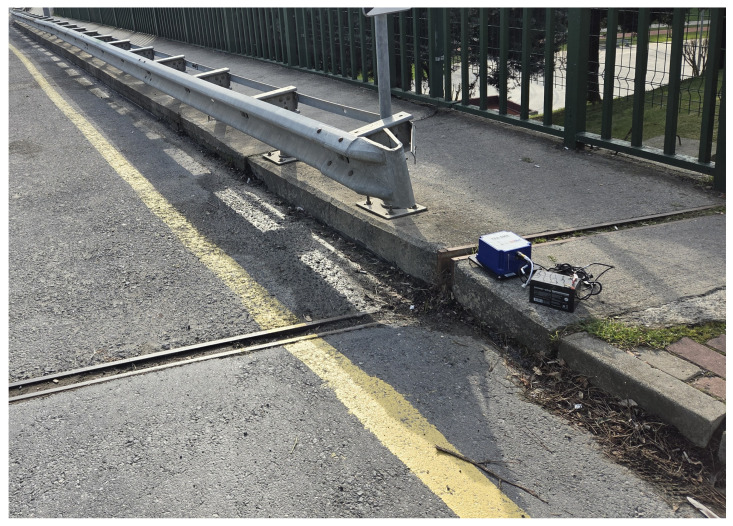
Hardware deployment during the ambient vibration tests. The high-sensitivity triaxial accelerometer (EQMet TSA-SMA) and data acquisition setup are positioned adjacent to the bridge expansion joint to effectively capture cross-span interaction dynamics under operational traffic loads.

To quantify the impact of the localized RC jacketing, two distinct Ambient Vibration Test (AVT) campaigns were executed: a Baseline Campaign (Damaged State) prior to retrofitting, and a Monitoring Campaign (Retrofitted State) conducted after the curing of the RC jacket. Data acquisition was performed using high-fidelity triaxial strong motion accelerographs (EQMet TSA-SMA). These force-balance sensors are equipped with a 24-bit internal digitizer and a broadband frequency response (DC to 225 Hz). This configuration provides a high dynamic range, enabling the accurate capture of low-amplitude ambient vibrations induced by operational traffic loads. The hardware deployment and the specific positioning of the sensor adjacent to the bridge expansion joint are depicted in Figure 2.

**Figure 3:**
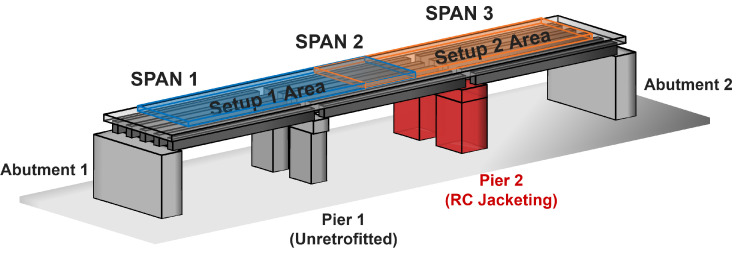
Schematic elevation view of the multi-span bridge, highlighting the location of Span 1, Span 2, Span 3, and indicating the specific roving sensor setups and the pier (Pier 2) where the RC jacketing was applied.

A sequential pairwise roving sensing strategy was implemented to address the strict hardware constraint of utilizing only two accelerometers (Figure 3). This configuration represents a highly realistic scenario for scalable and cost-effective real-world SHM deployments. The measurement campaign was divided into two independent spatial setups. Setup 1 recorded the dynamic interaction between Span 1 and Span 2, whereas Setup 2 captured the interaction across the retrofitted interface between Span 2 and Span 3.

It is crucial to note that strict time synchronization was ensured between the two sensors within each individual setup. While traditional OMA necessitates a fixed reference sensor across all setups to stitch global mode shapes together, the proposed data-driven framework intentionally bypasses this requirement. The proposed signal processing pipeline circumvents the need for absolute global time synchronicity. It achieves this by shifting the focus from global mode shape reconstruction to localized cross-span interaction dynamics and spatial energy transfer, utilizing ARX, WPD, and coherence metrics. This enables robust feature extraction directly from fragmented, non-synchronous sensor data, significantly reducing data transmission overhead.

The continuous recordings were sampled at a frequency of 200 Hz. While the accelerometers recorded triaxial data, only the vertical acceleration responses were utilized for the subsequent damage detection algorithms, as they contain the most dominant modal energy for traffic-induced excitations. These vertical responses were systematically segmented into non-overlapping moving windows of 20 s (N=4000 data points per window) and subjected to a detrending process to remove DC offsets. This segmentation process yielded a total of 158 observation windows for the baseline damaged state, and 1290 windows for the retrofitted monitoring state. Although the practical constraints of the field campaigns resulted in an imbalanced dataset, the proposed Mahalanobis-based condition assessment framework is inherently robust against this disparity. The condition index ($D_{M}$) solely requires a statistically representative baseline distribution to accurately construct the baseline covariance matrix (Σ_base_), meaning that an equal number of samples across the two structural states is not a mathematical prerequisite for highly reliable classification [2].

### 2.3. Hierarchical Multi-View Feature Fusion Framework

**Figure 4:**
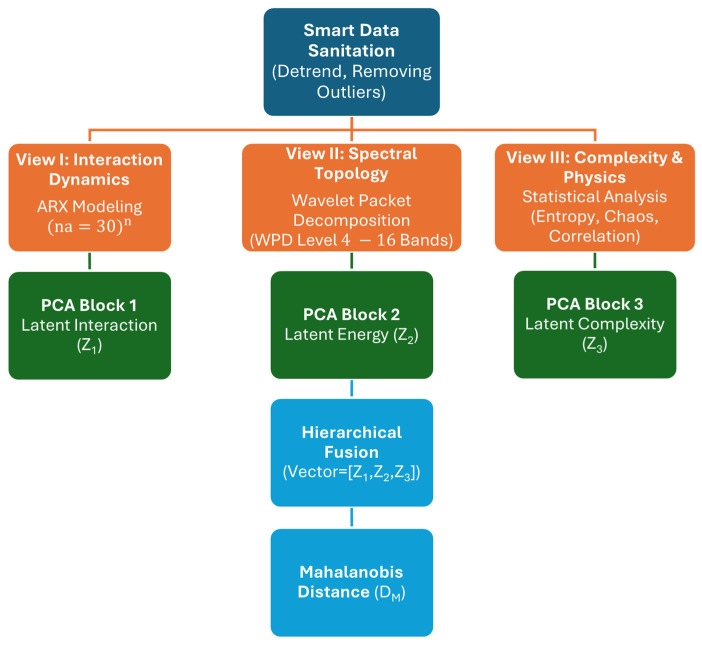
Comprehensive flowchart of the proposed data-driven SHM framework, detailing the sequence from raw ambient vibration acquisition to multi-view feature extraction (ARX, WPD, Statistics), PCA-based hierarchical fusion, and Mahalanobis distance quantification.

Vibration signals acquired from continuous bridge structures under ambient excitation are inherently non-stationary, highly dimensional, and frequently contaminated with environmental noise. To robustly quantify the structural regime shift, the raw vibration signals are partitioned, and three distinct “views” of the structural state are extracted: Interaction Dynamics, Spectral Topology, and Time-Domain Statistical Descriptors. The overall architecture of the proposed hierarchical multi-view feature fusion framework is systematically illustrated in Figure 4.

#### 2.3.1. View I: Latent Interaction Dynamics via High-Order ARX Modeling

In the context of multi-sensor data fusion, time-series modeling via Autoregressive with eXogenous inputs (ARX) is deployed as an autonomous feature extractor to decode the cross-channel vibration transmission directly from the raw time-series data. Let u(t) represent the ambient vibration response of the reference span and y(t) represent the response of the adjacent span. The linear ARX model is expressed as:

(1) $$y\left(t\right)+\;\sum_{i=1}^{n_{a}}a_{i}\;y\left(t-i\right)=\;\sum_{i=1}^{n_{b}}b_{j}\;u\left(t-j-n_{k}\right)+\;e\left(t\right)$$

where $n_{a}$ and $n_{b}$ are the orders of the autoregressive and exogenous polynomials; $n_{k}$ is the pure time delay; $a_{i}$ and $b_{j}$ are the unknown model coefficients; and $e\left(t\right)$ is the residual error. To adequately capture the wide-band frequency content and the complex, high-frequency spatial interactions inherent to ambient traffic excitations, a high-order model ($n_{a}=\;30$, $n_{b}=\;30$, $n_{k}=\;1$) was selected. The model order was systematically determined through preliminary parametric evaluations based on the minimization of the Akaike Information Criterion (AIC) and residual analysis. This sensitivity-based justification ensures that the extracted features are stable across varying operational windows, capturing the essential dynamics without overfitting. This specific high-order configuration ensures that the complex transient dynamics of the continuous spans are effectively embedded within the coefficients without inducing severe computational overfitting [26,27]. The estimated coefficients are concatenated to construct the first independent feature block, $F_{ARX}$, defined as a 1×60 dimensional feature vector:

(2) $$F_{ARX}=\;\left[a_{1},\;\ldots ,\;a_{\left\{30\right\}},\;b_{1},\;\ldots ,\;b_{\left\{30\right\}}\right]$$

This high-dimensional vector effectively embeds the transient interaction dynamics and vibration transmission characteristics of the bridge pier interface within its 60 dimensions.

#### 2.3.2. View II: Spectral Topology via Wavelet Packet Decomposition (WPD)

To map the multi-resolution spectral energy topology of the non-stationary response, WPD is utilized. The signal is decomposed using a Daubechies 4 (db4) mother wavelet at a decomposition level of j=4, yielding 16 terminal frequency bands. The db4 wavelet was specifically selected because its mathematical properties offer an optimal balance between orthogonality and compact support, making it highly effective for localizing the transient shocks and short-duration impact energies typical of traffic-induced bridge vibrations [21]. The selection of the ‘db4’ mother wavelet and a 4th-level decomposition was optimized through preliminary parameter sensitivity tests. Compared to alternative configurations—including Symlet (sym4) and Coiflet (coif3) families—the proposed db4 setup yielded the maximum structural separation margin and the lowest false positive rate, thereby confirming its optimality for isolating the fundamental dynamic bands of the bridge. The spectral energy $E_{\left\{j,n\right\}}$ of the *n*^th^ node is computed by summing the squared wavelet packet coefficients $d_{\left\{j,n\right\}\left(k\right)}$:

(3) $$E_{j,n}=\;\sum_{k}\begin{array}{c} {\left|d_{j,n}\left(k\right)\right|}^{2}\;\; \end{array}$$

To ensure immunity against varying ambient excitation amplitudes, the energy of each node is normalized by the total spectral energy:

(4) $$P_{j,n}=\frac{E_{j,n}}{\sum_{1}^{16}E_{j,n}}\times 100$$

The resulting 16 normalized energy percentages are organized into the second independent feature block, $F_{WPD}$, defined as a 1×16 dimensional feature vector:

(5) $$F_{WPD}=\;\left[P_{j,1},\;P_{j,2},\;\ldots ,\;P_{j,16}\right]$$

where each element $P_{j,n}$ represents the normalized spectral energy of the nth terminal node at the 4th decomposition level. This vector provides a comprehensive mapping of the structural response’s spectral topology, capturing energy shifts across the entire frequency range.

#### 2.3.3. View III: Time-Domain Statistical Descriptors

To quantify the physical intensity and complexity of the vibration time-series, a set of Time-Domain Statistics (TDS) is extracted: Root Mean Square (RMS), Variance, Skewness, and Kurtosis. Furthermore, Hjorth Mobility (M) is calculated to capture signal complexity:

(6) $$M=\frac{\sigma_{\dot{y}}}{\sigma_{y}}$$

where $\sigma_{y}$ and $\sigma_{\dot{y}}$ are the standard deviations of the signal’s first derivative and the signal itself, respectively. For each observation window, these five statistical indicators are concatenated to construct the third independent feature block, $F_{TDS}$, defined as a feature vector:

(7) $$F_{TDS}=\;\left[RMS,\;\sigma^{2},\;S,\;K,\;M\right]$$

where $\sigma^{2}$ is the variance, S is the skewness, K is the kurtosis, and M is the Hjorth Mobility. This vector essentially compresses the time-domain physical intensity and complexity of the signal into a 1×5 dimensional space before the fusion process.

#### 2.3.4. Hierarchical Feature Fusion and Damage Quantification

To construct a comprehensive structural condition index, the three independent feature blocks F*_ARX_*, F*_WPD_*, and F*_TDS_* are first integrated into a high-dimensional master matrix and subjected to Z-score standardization to ensure zero mean and unit variance across all dimensions.

To eliminate redundant information and isolate the deterministic structural response from stochastic noise, a block-wise Principal Component Analysis (PCA) strategy is implemented. Instead of performing PCA on the entire concatenated set, the algorithm treats each feature domain as a separate physical “view”. Because the PCA operates exclusively on these time-independent statistical features rather than the raw non-synchronous waveforms, issues such as time-domain spatial distortion and mode aliasing across different setups are fundamentally bypassed. This block-wise PCA strategy was explicitly implemented to address potential multicollinearity within the high-dimensional feature space. By projecting each domain independently, redundant information and cross-correlations between different physical descriptors are eliminated. Each standardized block is individually filtered to extract its first principal component (PC1), which captures the maximum variance and serves as the most sensitive latent representative of that specific domain while effectively suppressing Environmental and Operational Variations (EOVs). The principal components for each feature domain are extracted by mapping the high-dimensional blocks into their respective first principal components (PC1):

(8) $$Z_{k}=\;PCA_{PC1}\left(F_{k}\right),\;\mathrm{for}\;k\in \;\{ARX,\;WPD,\;TDS\}$$

where $Z_{k}$ represents the latent representative of the corresponding physical view. These three distinct latent variables are subsequently concatenated to form the final 3-Dimensional Latent Feature Space vector, V:

(9) $$V\;=\;\left[Z_{ARX},\;Z_{WPD},\;Z_{TDS}\right]$$

Finally, the Mahalanobis distance ($D_{M}$) is employed to statistically quantify the structural regime shift within this fused latent space. Operating as an unsupervised statistical anomaly detection mechanism, this approach does not rely on labeled training datasets or traditional classification accuracy metrics, making it highly suitable for real-world SHM where damaged state data is often unavailable. By accounting for the covariance between the fused variables, the condition index $D_{M}$ for any observation vector x is calculated as:

(10) $$D_{M}\left(x\right)=\sqrt{{\left(x-\mu_{base}\right)}^{T}\Sigma_{base}^{-1}\left(x-\mu_{base}\right)}$$

where $\mu_{base}$ and ∑_base_ are the mean vector and covariance matrix of the baseline (damaged) state, respectively. While the Mahalanobis distance is a robust metric for multivariate outlier detection, it is important to acknowledge that related statistical approaches, such as Hotelling’s $T^{2}$ statistic, also offer powerful capabilities for process control and robust condition estimation in complex SHM datasets [3]. To establish a rigorous decision boundary, a 99% statistical confidence threshold is computed using the inverse Cumulative Distribution Function of the Chi-Square ($\chi^{2}$) distribution with three degrees of freedom.

## 3. Results

This section presents the comprehensive evaluation of the localized retrofitting intervention on the global structural dynamics. The analysis is presented in three progressive stages: first, the preliminary physical assessment of vibration attenuation; second, the evaluation of the structural regime shift via spectral and spatial synchronization metrics; and finally, the visual and statistical validation in a data-driven multi-view latent feature space.

### 3.1. Spectral Evolution and Spatial Decoupling

**Figure 5:**
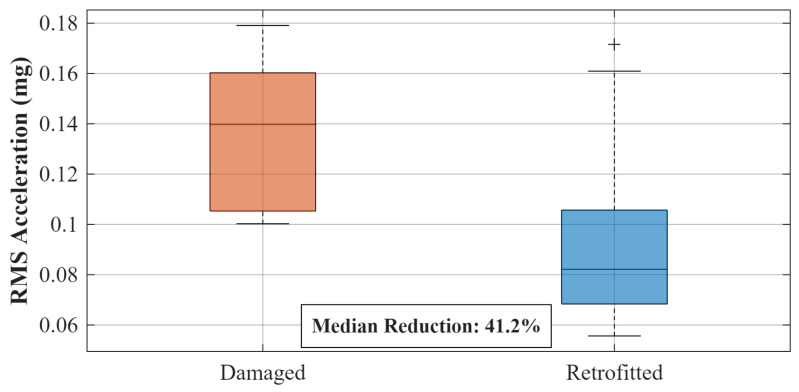
Statistical box plot comparing the Root Mean Square (RMS) acceleration (in mg) of the bridge under ambient loads. The retrofitted state demonstrates a significantly tighter variance and a 41.2% reduction in median vibration intensity (dropping from approximately 0.14 mg to 0.08 mg), confirming global stiffening. The ‘+’ symbol in the plot represents an outlier data point.

Prior to evaluating the complex cross-span spatial interactions, a fundamental time-domain assessment was performed to quantify the macroscopic stiffening effect induced by the localized intervention. As a robust indicator of the overall kinetic energy, the Root Mean Square (RMS) acceleration was computed to measure the physical vibration amplitude of the bridge under ambient operational loads. Figure 5 presents the statistical distribution of these RMS values (in mg) extracted from all observation windows for both the damaged and retrofitted states.

**Figure 6:**
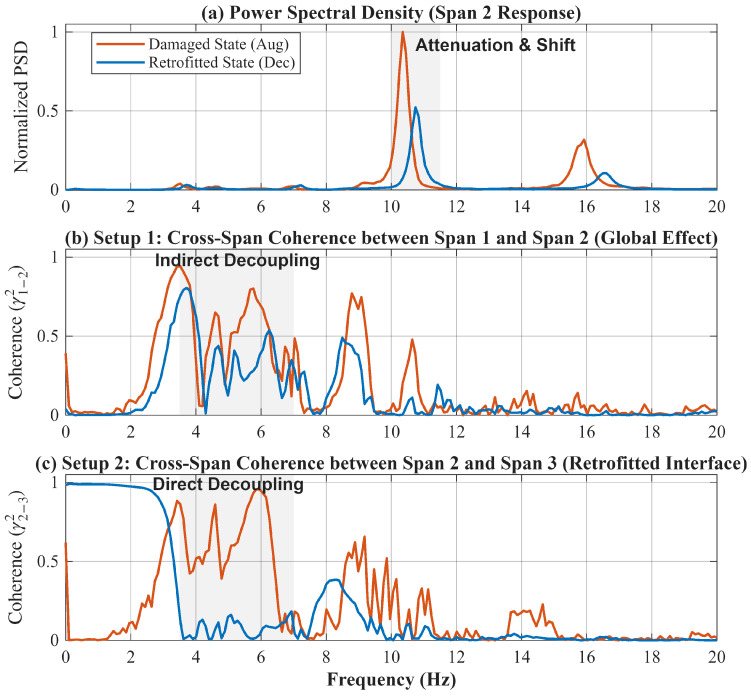
Spectral evolution and spatial synchronization of the bridge before and after the retrofit intervention. The grey shaded regions highlight the specific frequency bands where these primary structural changes are most prominent. (**a**) Normalized Power Spectral Density (PSD) showing the attenuation of vibration energy and the shift in resonance frequency (stiffening effect). (**b**) Averaged cross-span coherence between Span 1 and Span 2 computed over all observation windows of Setup 1, illustrating the indirect decoupling effect. (**c**) Averaged cross-span coherence between Span 2 and Span 3 from Setup 2, showing direct decoupling at the retrofitted interface. The comparison between the damaged and retrofitted states relies on the statistical distributions of coherence obtained under similar ambient conditions.

Conventional signal processing techniques, specifically Power Spectral Density (PSD) and cross-sensor magnitude-squared coherence ($\gamma^{2}$), were initially employed to establish a physical understanding of the structural changes before and after the RC jacketing. The spectral evolution and spatial synchronization across the continuous spans are illustrated in Figure 6.

The PSD estimates presented in Figure 6a reveal a distinct change in the fundamental resonance behavior of the structure. During the baseline (damaged) phase, the structure exhibited a sharp, high-energy peak centered around 10.3 Hz. This elevated amplitude is highly indicative of structural looseness, where the degraded pier interface failed to dissipate operational vibration energy efficiently. Following the localized RC jacketing of Pier 2, the monitoring data demonstrates a significant attenuation of this vibration energy. Furthermore, the primary resonance peak distinctly migrated rightward to approximately 10.8 Hz. According to fundamental structural dynamics, this frequency increase is the classical signature of a stiffness enhancement (stiffening effect) directly imparted by the RC jacket.

While the PSD confirms local stiffening, the primary advantage of the intervention is revealed through the cross-span spatial coherence ($\gamma^{2}$), which quantifies the dynamic coupling (energy transfer) between adjacent spans. Considering the average coherence spectra obtained from Setup 2 (Figure 6c), which captures the directly retrofitted interface, the low-frequency band (0–3.5 Hz) of the retrofitted state exhibits near-perfect coherence (γ^2^ ≈ 1.0). This indicates that, on average, the two spans respond as a monolithic rigid body under global quasi-static loads, confirming the stiffening effect of the RC jacket. Conversely, in the critical high-energy resonance band (4–7 Hz), the damaged state exhibited elevated coherence (approaching 0.95), meaning the loose spans were interacting irregularly and transferring high-frequency vibration energy across the degraded joint. Post-retrofit, the coherence in this specific band decreases significantly to near zero. The RC jacketing significantly increased the flexural stiffness of Pier 2, essentially transforming it into a rigid dynamic barrier.

A clear indication of global stabilization emerges from Setup 1 (Figure 6b), which shows the coherence between Span 1 and Span 2—an interface that received no direct intervention. Interestingly, the strong dynamic coupling previously observed in the 4–7 Hz band (damaged state) almost completely disappears in the retrofitted state. We term this phenomenon *indirect decoupling*: the localized stiffening of Pier 2 alters the boundary condition of Span 2, which in turn isolates Span 1 from the high-frequency vibration leakage.

To rigorously distinguish the effects of the localized RC jacketing from Environmental and Operational Variations (EOVs), specifically seasonal temperature changes, the ambient conditions during the baseline and monitoring campaigns were recorded. The ambient temperature during the August baseline ranged between 25 °C and 30 °C, whereas the December monitoring campaign was conducted under significantly colder conditions, ranging from 5 °C to 10 °C. A temperature decrease of this magnitude typically induces a global increase in the concrete’s elastic modulus. This accounts for a partial contribution to the observed rightward frequency shift from 10.3 Hz to 10.8 Hz [6]. However, thermal stiffening alone cannot physically justify the 41.2% reduction in median RMS acceleration. Environmental effects generally induce gradual, low-amplitude variance in vibration intensity rather than abrupt, permanent scale drops [9,28]. Furthermore, global thermal variations inherently affect the entire continuous structure uniformly. They cannot cause the localized cessation of dynamic cross-span coupling (the coherence drop to near-zero in the 4–7 Hz band) strictly at the retrofitted interface. Therefore, while EOVs marginally contributed to the global frequency baseline shift, the distinct spatial decoupling and vibration attenuation effectively identify the localized RC jacketing as the main driver for the structural regime shift.

### 3.2. 3D Latent Feature Space Representation

**Figure 7:**
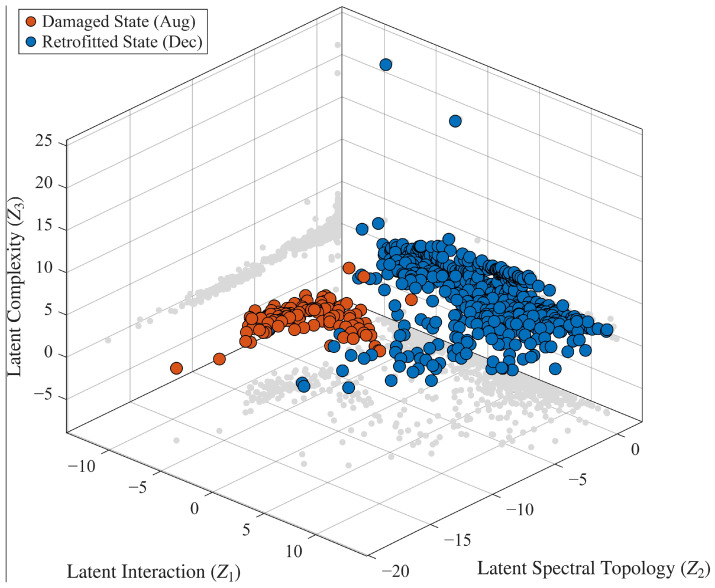
3D Multi-View Feature Fusion Space representing the structural regime shift. The latent coordinates correspond to Interaction Dynamics ($Z_{1}$), Spectral Topology ($Z_{2}$), and Complexity ($Z_{3}$). The explicitly separated clusters (with zero overlap) and their orthogonal shadow projections visually validate the success of the hierarchical feature extraction in distinguishing the damaged baseline from the retrofitted state. The light grey dots on the bounding planes indicate these orthogonal shadow projections, representing the 2D projections of the 3D data points onto the coordinate planes.

While the spectral and coherence analyses provide crucial physical insights into the vibration attenuation and spatial decoupling, relying on individual signal processing metrics can be insufficient to capture the full dimensionality of a structural regime shift. To comprehensively visualize the global impact of the localized RC jacketing, the extracted multi-view features—Interaction Dynamics, Spectral Topology, and Time-Domain Statistical Descriptors—were fused into a compact, low-dimensional space using Principal Component Analysis (PCA). The resulting 3D latent feature space is constructed using the first three principal components ($Z_{1}$, $Z_{2}$, and $Z_{3}$) as shown in Figure 7.

As depicted in Figure 7, the observation windows from the damaged state and the retrofitted state form two strictly mutually exclusive clusters. There is distinct spatial separation with no overlap observed within the evaluated datasets between the two structural states. This distinct spatial separation mathematically confirms that the RC jacketing did not merely cause minor local frequency fluctuations, but rather induced a fundamental global transformation in the bridge’s dynamical topology.

Beyond the clear separation, the geometric characteristics of the clusters provide valuable information regarding the structural behavior. The damaged baseline cluster exhibits a relatively scattered and spherical variance, representing the chaotic, loose, and highly uncertain dynamic responses of the degraded structure under varying traffic loads. Conversely, the retrofitted cluster is highly compact and elongated along a specific directional axis. This geometric tightening in the latent space indicates that the structural behavior has transitioned from a stochastic, loose state to a highly deterministic and rigid state. Furthermore, the robustness of this multi-view fusion is visually reinforced by the orthogonal shadow projections on the 2D boundary planes, demonstrating that a simple machine learning classifier could effectively separate the damaged and retrofitted conditions with high precision, without any false classifications.

### 3.3. Statistical Quantification via Mahalanobis Distance

**Figure 8:**
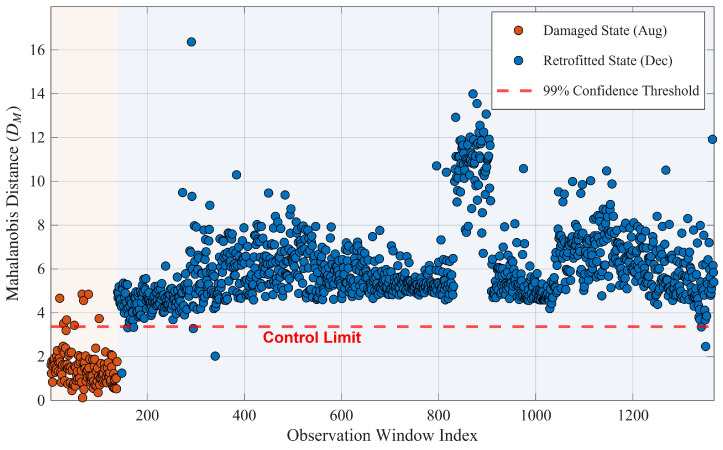
Mahalanobis distance ($D_{M}$) control chart illustrating the statistical quantification of the structural regime shift. The dashed red line represents the 99% confidence control limit. Note: To better illustrate the data distribution near the control limit, the *y*-axis is capped at a $D_{M}$ value of 30, omitting a few extreme outliers in the retrofitted state that reached up to $D_{M}=\;70$.

Although the 3D latent feature space provides clear qualitative visualization of the structural regime shift, reliable Structural Health Monitoring (SHM) requires statistical quantification to rule out random data variance and environmental noise. To achieve this, the Mahalanobis distance ($D_{M}$) was computed to convert the 3D spatial separation into a normalized, one-dimensional structural condition index. The Mahalanobis distance control chart is calculated using the damaged state as the reference baseline distribution (Figure 8). To establish a rigorous decision boundary, a 99% statistical confidence threshold was computed based on the inverse Cumulative Distribution Function of the Chi-Square ($\chi^{2}$) distribution with three degrees of freedom.

As observed in the baseline phase of Figure 7, all measurements from the damaged state naturally fluctuate below or strictly near the 99% control limit. This baseline stability validates the accuracy of the established $\chi^{2}$ threshold, indicating zero false-positive alarms under normal operational variations. However, when the data from the retrofitted monitoring phase is evaluated against this baseline distribution, the system exhibits a significant and distinct increase. The $D_{M}$ values increase sharply, consistently ranging between 5 and 20, with certain traffic-induced peak events (e.g., the passage of heavy commercial vehicles) reaching values as high as 70. Most importantly, the $D_{M}$ values of the retrofitted state never drop back below the control limit.

This sustained, substantial deviation strongly indicates that the detected change is not a temporary anomaly or an environmental artifact, but a permanent, irreversible structural modification. The Mahalanobis metric mathematically validates that the localized RC jacketing on Pier 2 successfully stabilized the structure, clearly separating the system’s operational states and demonstrating a persistent structural regime shift with over 99% statistical confidence.

## 4. Discussion

The findings of this study demonstrate that localized structural interventions, such as RC jacketing, can be robustly evaluated using ambient vibration data when processed through a hierarchical multi-view feature fusion framework. Traditionally, the SHM community has relied heavily on global frequency shifts to assess structural changes [8,9]. However, as observed in our spectral analysis, frequency migration alone is often subtle and easily masked by Environmental and Operational Variations (EOVs). By shifting the focus to cross-span spatial coherence, our results provide a new perspective: localized stiffening does not merely alter frequencies but fundamentally modifies the global dynamical topology by inducing both direct and indirect spatial decoupling. This indirect decoupling mechanism overcomes the limitations of traditional linear modal analysis, offering a more sensitive indicator of structural boundary condition changes.

Furthermore, the integration of Interaction Dynamics (ARX), Spectral Topology (WPD), and Statistical Complexity into a fused 3D latent space aligns with recent advancements in machine learning for SHM [18,20]. Yet, this study extends previous works by proving that Block-wise PCA can act as a powerful physical noise filter. The distinct zero-overlap bifurcation observed between the damaged and retrofitted clusters visually and mathematically confirms that the proposed algorithm successfully isolates the deterministic structural regime shift from stochastic environmental noise. Furthermore, implementing a 99% confidence threshold integrates rigorous uncertainty quantification into the analytical pipeline. This ensures the detected shift is statistically significant (p < 0.01) and highly distinguishable from inherent measurement noise. Statistical validation using the Mahalanobis distance ($D_{M}$) at a 99% confidence interval provides a highly reliable, threshold-based condition index that eliminates the subjectivity of visual inspections.

Moreover, from a sensing architecture perspective, this study validates that a sparse, pairwise roving sensor setup can yield the same diagnostic depth as a permanently dense sensor array when coupled with the proposed multi-view data fusion pipeline. While a traditional fixed sensor array for a multi-span bridge of this scale would typically require 10 to 20 permanently installed nodes, the proposed framework achieves high-resolution spatial mapping using only two sensors, resulting in an estimated reduction of over 80% in hardware and installation costs. This strategy significantly mitigates extensive cabling requirements and data transmission bottlenecks, addressing the primary physical obstacles in scalable SHM deployments.

From a practical engineering perspective, the proposed framework offers significant computational efficiency. Unlike deep learning-based SHM architectures that require extensive training on high-performance GPUs, the proposed hierarchical fusion pipeline is numerically lightweight. Feature extraction and PCA projection for a 20-s observation window execute in fractions of a second on conventional CPUs, demonstrating its high feasibility for real-time edge-computing and scalable field deployments.

While the proposed framework demonstrates high reliability, the following limitations are noted. Primarily, since the physical asset is a fully operational public highway bridge, inducing progressive artificial damage scenarios to evaluate varying damage severities was strictly prohibited due to safety regulations, inherently restricting the field validation to a binary real-world event (pre- and post-retrofit conditions). The baseline measurement campaign was restricted to a specific seasonal window (August). Consequently, the established baseline covariance matrix may not fully encapsulate extreme annual temperature gradients or varying traffic mass extremes over a full calendar year. The absence of year-round synchronous temperature monitoring is a recognized limitation; however, by shifting the diagnostic focus from global frequencies to localized spatial features like Cross-Span Coherence, the proposed framework explicitly filters out global environmental noise. This spatial decoupling serves as a structural signature that is physically immune to seasonal thermal fluctuations. While the algorithm successfully isolated the structural stiffening from the measured December conditions, long-term continuous monitoring is necessary to build a comprehensively generalized seasonal baseline.

### 4.1. Discrimination of Retrofitting Effects from Thermal Variations

In structural health monitoring, decoupling Environmental and Operational Variations (EOVs) from permanent structural changes is a fundamental challenge. For the investigated prestressed concrete bridge, the baseline campaign was conducted in August (at an ambient temperature of approximately 25 °C), whereas the post-intervention monitoring occurred in December (at approximately 5 °C). Because the elastic modulus of concrete, $E\left(T\right)$, and the boundary stiffness of elastomeric bearings are inherently temperature-dependent, it is imperative to quantitatively evaluate whether the detected regime shift stems from the localized structural retrofitting or merely from seasonal thermal stiffening.

To establish the theoretical bounds of the environmental influence, a temperature sensitivity analysis was considered. According to landmark long-term SHM studies specifically conducted on multi-span prestressed concrete bridges [6,29], a decrease in ambient temperature induces an increase in global natural frequencies. For prestressed concrete structures, this thermal stiffening rate typically ranges between 0.20% and 0.50% per 1 °C drop. Over the measured 20 °C thermal differential in this study, bridge dynamics predict a theoretical frequency increase ranging from 4.0% to 10.0%. For the initial baseline fundamental frequency of 10.3 Hz, this environmental stiffening translates to a theoretical shift of roughly +0.40 to +1.0 Hz.

The actual observed global frequency shift of +0.5 Hz (a 4.85% increase, equating to ~0.24% per 1 °C, as discussed in Section 3) falls well within these theoretical thermal bounds for prestressed concrete bridges. This observation confirms that traditional global frequency tracking is highly susceptible to environmental contamination.

However, the proposed Block-wise PCA framework inherently addresses this limitation by abandoning global frequency tracking in favor of spatial feature evaluation. In structural dynamics, a global, uniform temperature drop predominantly scales the overall stiffness matrix, which shifts the system’s eigenvalues (frequencies). Yet, uniform cooling largely preserves the fundamental boundary conditions of the simply-supported spans, thereby maintaining the structural mode shapes and proportional inter-channel relationships (eigenvectors).

The proposed methodology utilizes PCA to project multi-view feature blocks onto their primary principal components, essentially extracting the spatial covariance structure of the localized feature space. While homogeneous thermal stiffening may uniformly scale the amplitude of the extracted time-series features (such as ARX coefficients or statistical moments), it mathematically preserves their underlying spatial covariance topology.

Consequently, the substantial anomaly detected by the Mahalanobis distance ($D_{M}$) metric significantly exceeds the theoretical bounds of a pure uniform temperature drop. The localized structural retrofitting physically modified the support boundary conditions, creating a distinct mechanical discontinuity that fundamentally distorted the spatial covariance of the dynamic response. By focusing on the variance within the feature space rather than absolute frequency values, the algorithm effectively filters out uniform thermal stiffening as low-variance background noise, thereby isolating the high-variance regime shift caused by the physical retrofitting.

### 4.2. Handling of Asynchronous Data and Scope of Application

The practical implementation of roving-sensor configurations in OMA introduces specific signal processing challenges, particularly regarding data synchronization and spectral ambiguities such as mode aliasing. The proposed Block-wise PCA framework addresses these limitations by shifting the analysis from continuous time-domain tracking to an anchored statistical feature space.

#### 4.2.1. Asynchronous Data Alignment via Covariance Mapping

Unlike conventional output-only modal identification techniques that necessitate strict phase synchronization or time-domain alignment across roving batches, the proposed methodology achieves alignment through a generalized covariance structure. By maintaining a single fixed reference sensor across all independent measurement campaigns, the multi-view features (ARX coefficients, Wavelet packet energies, and statistical moments) extracted from the roving nodes are intrinsically normalized against the baseline operational and environmental state. When the Block-wise PCA algorithm projects these localized feature blocks onto their first principal components, it aligns the underlying spatial covariance topology of the data rather than its temporal phase. This reference-anchored standardization eliminates the requirement for non-linear time-domain transformation techniques, such as Dynamic Time Warping (DTW), which risk introducing artificial phase distortions into non-stationary acceleration records [2].

#### 4.2.2. Mitigation of Mode Aliasing via Multi-Domain Fusion

Spatial and frequency-based mode aliasing represents a significant constraint in sparse monitoring networks, particularly when relying on conventional Fourier-based spectral peak-picking under closely spaced structural modes. The proposed framework mitigates this risk by expanding the diagnostic dimensionality through multi-domain feature fusion. While overlapping structural modes may alias within a pure frequency-domain representation, their distinct mechanical properties are preserved through complementary domains. The integration of ARX coefficients captures the underlying system poles and zeros—reflecting localized damping and phase variations—while WPD quantifies the transient energy dissipation rates across localized frequency sub-bands. By embedding these diverse signal descriptors into a unified high-dimensional matrix, the subsequent PCA identifies orthogonal directions of maximum variance. Consequently, the framework distinguishes structural regime shifts based on multi-dimensional statistical variance, bypassing the technical ambiguities associated with traditional modal frequency identification.

#### 4.2.3. Definition of Operational Scope and Boundaries

To ensure the reliable deployment of the framework, its engineering scope and boundaries must be explicitly defined. The Block-wise PCA approach is strictly engineered as a data-driven anomaly detection and early-warning framework tailored for structures monitored via sparse, asynchronous, or non-permanent sensor topologies. It is highly applicable for identifying localized mechanical discontinuities, boundary condition alterations (such as the discussed RC jacketing intervention), or sudden stiffness degradations where dense, synchronous permanent instrumentation is logistically or economically unfeasible. Conversely, the methodology is not designed for high-resolution 3D physical mode shape reconstruction, precise damage localization within uninstrumented structural components, or deterministic Finite Element Model (FEM) updating. Because the framework tracks statistical variations within a transformed feature space rather than physical eigenvectors, it serves as a robust indicator of structural regime shifts rather than a geometric diagnostic tool.

### 4.3. Quantitative Comparative Analysis and Robustness Evaluation

To verify the practical feasibility, algorithmic innovation, and reliability of the proposed Block-wise PCA framework, a comprehensive comparative ablation study was performed against mainstream data-driven alternatives: a Single-Feature model (utilizing time-domain ARX coefficients exclusively) and an Early Fusion approach (direct concatenation of ARX, WPD, and statistical features into an 81-dimensional vector). The monitoring performance was evaluated using the FDR under both baseline field conditions and severe operational anomalies, including measurement noise (20 dB SNR artificial Additive White Gaussian Noise (AWGN)) and intermittent sensor data loss. Table 2 summarizes the quantitative findings and the computational processing time monitored per 20-s observation window.

#### 4.3.1. Performance Under Standard and Noisy Conditions

As demonstrated in Table 2, the standard Early Fusion approach yields an extremely low FDR of 0.02 under clean conditions. This performance degradation stems from spatial distortion and the “curse of dimensionality” encountered when high-dimensional, non-synchronous feature blocks are directly concatenated without hierarchical alignment. Conversely, the proposed Block-wise PCA framework achieves a clean-data FDR of 2.45, establishing a separation margin substantially higher than direct concatenation.

Under a 20 dB noise interference scenario, the proposed framework maintains a dominant separation capability with an FDR of 9.04. This stability is fundamentally governed by the orthogonal projection mechanism of the PCA algorithm, which segregates high-frequency stochastic noise into lower-order, discarded principal components. By utilizing only the primary principal components for the Mahalanobis distance ($D_{M}$) calculation, the framework effectively filters out instrumentation noise while preserving the underlying structural covariance topology. Regarding computational efficiency, the proposed framework introduces virtually zero computational overhead (0.227 s) compared to standard Early Fusion, confirming its suitability for real-time edge-computing deployment.

#### 4.3.2. Robustness Against Sensor Data Loss

Beyond continuous measurement noise, wireless monitoring networks are highly susceptible to intermittent transmission failures or localized sensor faults, resulting in random data packet loss. Traditional structural identification methods that rely on continuous time-domain phase tracking are typically vulnerable to such temporal discontinuities. The proposed methodology circumvents this limitation through its localized, multi-domain feature extraction layer. Rather than requiring absolute signal continuity, the framework extracts statistical properties—such as low-order statistical moments and localized energy distributions via Wavelet Packet Decomposition (WPD)—from bounded observation windows.

In digital signal processing, random data packet loss (e.g., dropping 5% to 10% of data points within a defined time block) introduces localized amplitude variations but does not significantly alter the underlying probability density function (PDF) or the overall variance of that specific block. Because the extracted statistical moments and frequency-band energies remain stable despite missing samples, the resulting fused feature vector is preserved. Therefore, the subsequent Block-wise PCA process, which tracks the variance topology across this feature space, maintains its diagnostic reliability without triggering false anomaly alarms due to transient data gaps. This dual resilience against both continuous noise and discrete data loss supports the suitability of the framework for long-term, continuous engineering applications in harsh field environments.

### 4.4. Future Research Directions

While this study successfully validated the framework on a full-scale RC bridge undergoing localized jacketing, future research should explore its scalability. Testing the proposed multi-view fusion approach under varying degrees of damage severity and on different infrastructure typologies (e.g., steel or composite bridges) would further generalize its applicability. Additionally, integrating this algorithmic pipeline with real-time Internet of Things (IoT) sensor networks could facilitate the development of fully autonomous, cloud-based early warning systems for civil infrastructures.

To ensure the long-term stability, algorithmic reliability, and continuous edge-computing deployment of the proposed framework under real-world operational environments, the long-term monitoring strategy addresses three key operational requirements:

1. Computational Sustainability: As demonstrated in the execution timing analysis (Table 2), the hierarchical feature extraction layer introduces negligible computational overhead (0.227 s per observation window). This efficiency ensures that the algorithm can operate continuously on low-power, cost-effective edge-computing hardware installed permanently at the bridge site without requiring high-bandwidth data transmission or cloud-dependent processing.
2. Seasonal EOV Management: Environmental and Operational Variations (EOVs), particularly annual thermal cycles, introduce benign structural variance that can mask damage or trigger false anomalies. To mitigate this, the framework’s baseline spatial covariance topology will be coupled with an adaptive thresholding mechanism. By updating or correcting the baseline covariance matrix against seasonal temperature bounds, the system gradually absorbs long-term material aging while remaining sensitive to sudden, localized structural shifts.
3. Data Loss Resilience: Wireless sensor networks in harsh field conditions are inevitably subject to intermittent signal attenuation, power dips, or localized sensor faults, leading to data packet loss. The proposed methodology circumvents dependency on absolute signal continuity by processing bounded statistical observation windows rather than relying on continuous time-domain phase tracking. Consequently, the diagnostic stability is preserved, minimizing false alarms during transient data gaps.

## 5. Conclusions

This study proposed a novel, data-driven SHM framework to quantify the global structural stabilization induced by a localized RC jacketing intervention. By utilizing a roving accelerometer network and hierarchically fusing ARX, WPD, and time-domain statistical features via Block-wise PCA, the complex spatial interactions of a continuous bridge were successfully decoded. The analysis confirmed the physical rigidification through a significant vibration attenuation (a 41.2% reduction in median RMS acceleration) and clearly confirmed the occurrence of direct and indirect spatial decoupling across the spans. Validated by a 99% confidence limit using the Mahalanobis distance, the framework isolated true structural improvements from ambient noise. The robustness of the framework against EOVs is mathematically ensured through the block-wise PCA sanitation and localized spatial decoupling analysis. Together, these techniques effectively isolate true structural changes from ambient temperature fluctuations and traffic-induced noise. Consequently, this methodology provides a practical non-destructive evaluation tool for continuous monitoring, addressing the limitations of traditional frequency-shift techniques.

## Figures and Tables

**Table 1:** Summary of structural parameters and retrofitting specifics.

Category	Parameter	Description/Value
**STRUCTURAL** **GEOMETRY**	Total Length & Spans	3 spans (29.40 m + 30.00 m + 29.40 m) Carriageway width: 11.44 m
	Superstructure	120 cm deep precast prestressed I-girders with a 25 cm composite concrete deck
	Substructure (Abutments & Piers)	Conventional wall-type abutments Double-column frame piers with an original cross-section of 1.28 m × 4.50 m
**ORIGINAL** **MATERIAL** **PROPERTIES**	Existing Concrete & Steel	Concrete Grade: 34 MPa Steel Yield Strength: 420 MPa (S420a)
**RETROFITTING** **SPECIFICS**	Primary Intervention	25 cm Reinforced Concrete (RC) Jacketing applied to the pier columns and pier caps
	New Material Properties	Concrete Grade: 35 MPa Steel Yield Strength: 420 MPa (B420C)
	Purpose of Retrofit	To restrict excessive transverse displacement, increase lateral shear capacity, and enhance overall seismic robustness

**Table 2:** Quantitative performance comparison of feature extraction and fusion strategies.

Method	FDR for Clean Data	Processing Time (Sec/Window)	FDR Under 20 DB SNR
**SINGLE-FEATURE (ARX ONLY)**	0.00	0.198 s	7.81
**EARLY FUSION (DIRECT CONCATENATION)**	0.02	0.227 s	0.29
**PROPOSED (BLOCK-WISE PCA)**	2.45	0.227 s	9.04

## Data Availability

The raw data presented in this study are available on request from the corresponding author. The data are not publicly available due to privacy and security restrictions regarding the specific civil infrastructure asset investigated.

## Abbreviations

The following abbreviations are used in this manuscript:

ARX	AutoRegressive with eXogenous inputs
AVT	Ambient Vibration Test
EOV	Environmental and Operational Variations
ML	Machine Learning
OMA	Operational Modal Analysis
PCA	Principal Component Analysis
PSD	Power Spectral Density
RC	Reinforced Concrete
RMS	Root Mean Square
SHM	Structural Health Monitoring
WPD	Wavelet Packet Decomposition
